# Beyond Demographics: Quantifying Structural Delay in COVID-19 Vaccine Uptake via Population-Scale Network Embeddings

**DOI:** 10.23889/ijpds.v11i4.3817

**Published:** 2026-07-06

**Authors:** Hekmat Alrouh, Tom Oreel, Tom Emery

**Affiliations:** 1 Erasmus University Rotterdam, Netherlands

Demographic models of COVID-19 vaccine uptake explain only part of the observed variation in vaccination timing, leaving substantial heterogeneity among individuals with similar socioeconomic profiles. This study examines whether population-scale social network structure accounts for part of this demographically unexplained variation. Using administrative microdata from Statistics Netherlands, we construct a nationwide multiplex network of approximately 13 million adults, linking household, family, school, workplace, and neighborhood ties. We first estimate a Cox proportional hazards model of time-to-vaccination including key demographic covariates and extract deviance residuals to operationalize deviations from demographic expectations. We then generate 32-dimensional node embeddings using DeepWalk to capture latent structural features of the multiplex network. Finally, we train an XGBoost model to assess whether network embeddings improve out-of-sample prediction of residual vaccination delay. Sensitivity analyses address potential under-ascertainment in the vaccination registry due to incomplete consent. By integrating survival modeling and representation learning at population scale, the study evaluates whether structural network position contributes to heterogeneity in vaccine adoption timing beyond traditional demographic predictors.

